# A Meta-Analysis on the Relations between EGFR R521K Polymorphism and Risk of Cancer

**DOI:** 10.1155/2014/312102

**Published:** 2014-10-21

**Authors:** Yinsheng Wang, Lidan Zha, Dan Liao, Xiaozhi Li

**Affiliations:** Department of Stomatology, The First Affiliated Hospital of Chongqing Medical University, Chongqing Medical University, No. 1 Yixueyuan Road, Chongqing 400016, China

## Abstract

The EGFR R521K polymorphism has been shown to reduce the activity of EGFR; however, the association between EGFR R521K polymorphism and the risk of cancer remains inconclusive; therefore we performed a meta-analysis to evaluate the relationship between EGFR R521K polymorphism and susceptibility to cancer. Our results suggest that the EGFR R521K polymorphism is not associated with risk of cancer, but the different chemosensitivity to anticancer drugs may need further investigation.

## 1. Introduction

Cancer is one of the leading causes of death in humans worldwide. It is not a single, well-defined, but a generalized term of heterogeneous diseases including more than a hundred types. Although each type presents an independent feature, the essential pathological characteristics are referred to as aberrant cell proliferation, invasion, and migration.

Many studies have investigated the role of human epidermal growth factor receptor (EGFR, also known as HER1 or ErbB1) in cancer. Abnormal expression or activity of EGFR observed in many types of cancer, like breast cancer, colorectal cancer, non-small-cell lung cancer, head and neck squamous cell carcinoma, has been considered as an important marker in tumorigenesis, which resulted in more aggressive tumor phenotype, higher recurrence rate, and poorer prognosis [1–4].

EGFR plays a crucial role in cancer because it is a receptor for a wide variety of ligands including amphiregulin, betacellulin, EGF, epigen, epiregulin, heparin binding EGF-like growth factor (HB-EGF), and type *α* transforming growth factor (TGF-*α*). The binding of ligand triggers the activation of MAPK, PI3K/Akt/mTOR, and JAK/STAT downstream signalling pathway, which are responsible for regulation of numerous tumorigenic functions such as cancer cell proliferation, antiapoptosis, and metastasis [5–8].

The EGFR R521K polymorphism, also termed rs11543848, rs2227983, R497K, or 142285G>A, has a guanine [G] to adenine [A] mutation leading to an arginine [R] to lysine [K] substitution at codon 521 located in the CR2 domain and resulted in an attenuated activity of the receptor [9, 10]. Several reports have explored the involvement of R521K polymorphism in risk of cancer, but these reports presented inconclusive and controversial results. Therefore, we conducted a meta-analysis on the published studies to evaluate the effect of EGFR R521K polymorphism with the risk of cancer.

## 2. Material and Methods

### 2.1. Publication Retrieval

A comprehensive search in PubMed, Web of Science, OVID, and Cochrane Library regarding R521K polymorphism was performed. The following search words were used: “EGFR,” “ErbB1,” “HER1,” “polymorphism,” “R497K,” “R521K,” “rs11543848,” “rs2227983,” “142285G>A,” and “cancer.” We also traced the references of the articles and reviews to include the potentially eligible original reports.

### 2.2. Inclusion and Exclusion Criteria

All articles were included in the meta-analysis if they were (1) case-control studies, (2) studies on the relationship of R521K polymorphism and cancer risk, (3) sufficient data for the frequencies of alleles and genotypes in cases and controls.

Articles were excluded if they were (1) not related to cancer, (2) of insufficient information, (3) animal, bacteria, or cell line studies, (4) studies on the basis of family or twins rather than random general ones, and (5) repeatedly published data.

### 2.3. Data Extraction

Two investigators extracted the information independently including first author, journal, year of publication, country of origin, cancer type, genotyping method, sample size, source of control groups, frequency of genotypes and alleles in cases and controls, and the Hardy-Weinberg equilibrium (HWE) of genotype distribution in controls. Any disagreements between the two investigators were resolved by consensus.

### 2.4. Statistical Analysis

HWE that has not been reported in the literatures was assessed with the De Finetti program (http://ihg.gsf.de/cgi-bin/hw/hwa1.pl). The odds ratios (ORs) and the corresponding 95% confidence intervals (CIs) were used to assess the association between the R521K polymorphism and cancer risk. The pooled ORs were performed in allelic genetic model (A versus G), codominant model (AA versus GG and GA versus GG), recessive genetic model (AA versus GA + GG), and dominant genetic model (AA + GA versus GG). Subgroup analyses were also conducted based on ethnicity and cancer type. Heterogeneity between studies was checked by Chi-square based* Q* statistics, and the *I* ^2^ index which expresses the percentage of the total variation across studies was also tested. If the *P* value was <0.05 or the *I* ^2^ index was ≥50%, the random-effect model was adopted; otherwise the fixed-effect model was adopted [24]. Publication bias was evaluated using Begg's funnel plots and Egger's test [25, 26]. Statistical analyses were performed with Review Manager (version 5.2; Cochrane Collaboration, Oxford, UK) and Stata software (version 12; Stata Corporation, College Station, TX, USA).

## 3. Results

### 3.1. Characteristics of Included Studies

**Figure 1:**
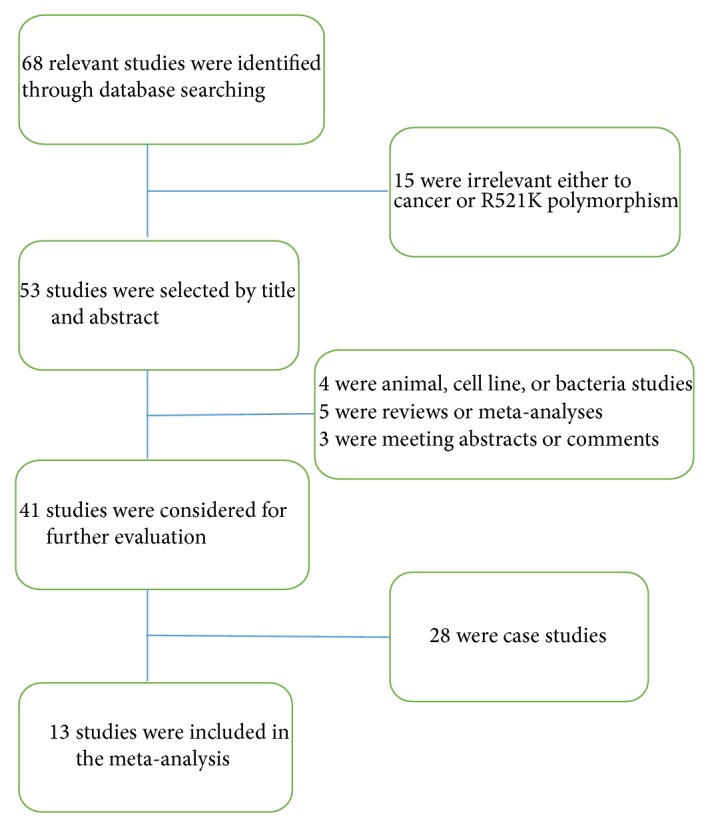
The flow diagram of the identification of studies.

After a thorough scan of the databases, 68 eligible studies related to EGFR R521K polymorphism were identified. 30 of them were initially excluded by title or abstract; 25 of them were excluded due to case studies. As a consequence, 13 studies containing 7328 cases and 8455 controls were included in this meta-analysis (Figure 1). Among the 13 studies, 4 studies were related to breast cancer [12, 20, 22, 23], 3 studies were related to colorectal cancer [11, 18, 21], 2 studies each were related to gastric carcinoma and hepatocellular cancer [13, 14, 16, 17], and each related to lung cancer and thyroid cancer [15, 19]. The ethnicities of subjects in 3 studies were Arabs and Caucasian, respectively; 5 studies were Asian, 1 study was Mexican and Indian, respectively. Nine studies recruited controls based on population, 3 studies recruited controls with respect to gender and age of cases, and 1 study took subjects as controls in population based and matching-criteria based manner. The distributions of the R521K polymorphism genotype in all control groups of the studies were in accordance with HWE. Detailed characteristics of the selected studies were shown in Table 1.

### 3.2. Quantitative Synthesis

The summary of the results for the association between EGFR R521K polymorphism and cancer susceptibility was shown in Table 2. In the overall analysis, no association between R521K polymorphism and cancer risk was found in all genetic models (A versus G: OR = 1.06, 95% CI: 0.95, 1.18, *P*_*H*_ < 0.0001, *I* ^2^ = 69, and *P* = 0.28; AA versus GG, OR = 0.85, 95% CI: 0.74, 0.99, *P*_*H*_ = 0.25, *I* ^2^ = 19, and *P* = 0.12; AA versus GA + GG: OR = 0.99, 95% CI: 0.91, 1.08, *P*_*H*_ = 0.84, *I* ^2^ = 0, and *P* = 0.80; GA versus GG: OR = 1.04, 95% CI: 0.87, 1.25, *P*_*H*_ < 0.00001, *I* ^2^ = 74, and *P* = 0.67; and AA + GA versus GG: OR = 1.02, 95% CI: 0.86, 1.21, *P*_*H*_ < 0.0001, *I* ^2^ = 73, and *P* = 0.84). In subgroup analysis, only a significant association between R521K polymorphism and reduced cancer risk was observed in the allelic genetic model in gastric cancer, but not in other genetic models (A versus G: OR = 1.43, 95% CI: 1.19, 1.72, *P*_*H*_ = 0.49, *I* ^2^ = 0, and *P* = 0.0001). No significant difference in other types of cancer and ethnicities was found.

### 3.3. Sensitivity and Metaregression Analyses

There was only low between-study heterogeneity in homozygote model (AA versus GG) and recessive genetic model (AA versus GA + GG) both in overall and subgroup analyses, but high heterogeneity was observed in allelic genetic model (A versus G), heterozygote model (GA versus GG), and dominant genetic model (AA + GA versus GG). Therefore the sensitivity analysis and metaregression analysis were performed to clarify the source of heterogeneity. In the sensitivity analysis, a single study was omitted each time to investigate the influence of the individual data on the pooled ORs. No significant alteration occurred in this procedure. In the metaregression analysis, neither cancer type nor ethnicity could explain the significant between-study heterogeneity.

### 3.4. Publication Bias

The results of the Begg funnel plots indicated no evidence of obvious asymmetry. The results of Egger's test also suggested little evidence of publication bias in the overall analysis (A versus G: *P* = 0.285; AA versus GG: *P* = 0.691; AA + GA versus GG: *P* = 0.713; AA versus GA + GG: *P* = 0.419; and GA versus GG: *P* = 0.319).

## 4. Discussion

It is well documented that the genetic polymorphism may contribute to susceptibility to cancer. In the current study, we reviewed all literatures regarding EGFR R521K polymorphism in cancer and conducted a meta-analysis to identify the association between the R521K polymorphism and the risk of cancer. The results revealed that no significant association was observed between R521K polymorphism and risk of cancer, except a statistical difference between A and G allele frequency in gastric cancer. The difference may be of low statistical power in view of the circumstance that there were only two articles related to gastric cancer.

EGFR is a member of the ErbB transmembrane receptors tyrosine kinase (RTK) family, whose members share a highly conserved extracellular ligand binding domain, a single-helix transmembrane segment, a tyrosine kinase juxtamembrane segment, and a regulatory carboxyterminal tail. The extracellular region of EGFR is composed of four domains: Domains I (L1), II (S1 or CR1), III (L2), and IV (S2 or CR2). L1 and L2 are members of the leucine-rich repeat family and are responsible for ligand binding. CR1 and CR2 are homologous cysteine-rich domains. In the absence of ligand, a ~25 Å *β*-hairpin loop extends from CR1 to contact the juxtamembrane region of CR2 and forms a tether structure. When ligand binds, the CR1/CR2 complex separates and the L1/CR1 composes a new binding site [27, 28]. The CR1/CR2 complex plays a role in determining the relative position of the EGFR dimers during ligand binding [29].

The R521K polymorphism is located in the CR2 domain and presents a lower affinity to TGF-*α*, a ligand which is related to cell proliferation, differentiation, and invasion after binding to EGFR. However, another study demonstrated that the CR1/CR2 tether structure exerted only a limited effect on EGF binding, EGFR activation, and cell signalling [30]. Take our result into consideration, we infer that the R521K polymorphism of EGFR may not be a critical factor in determining susceptibility to cancer; this conclusion is consistent with another meta-analysis indicating that the R521K polymorphism is not associated with risk of breast cancer [31]. Nevertheless, several studies have reported that the R521K polymorphism alone or in combination with a longer (CA)_*n*_ repeat polymorphism is associated with a better survival rate in cancer [32–36], while others report a negative relevance [37]. Furthermore, some studies have reported that the R521K polymorphism is associated with favorable outcomes in cetuximab-based and 5-FU-based chemotherapy [32, 35, 38–41] but shows negative correlation in gefitinib-based chemotherapy [37, 42, 43]. Not only indicate these results a complex mechanism in gene-gene interaction in regulating the function of EGFR, but also the different profiles of clinical response to chemotherapy suggest that the R521K polymorphism may be involved in the field of pharmacogenetics. In addition, the results also adduce a new evidence for the evolutionary mechanism of cancer; that is, single nucleotide polymorphism in individual gene, whether it would affect cancer cell proliferation or not, may exercise only a little influence on chromosome instability (CIN), and for this reason, the cancer progression is not altered remarkably [44, 45].

Studying heterogeneity was a considerable problem in our meta-analysis. The heterogeneity existed in allelic genetic model (A versus G), heterozygote model (GA versus GG), and dominant genetic model (AA + GA versus GG) in overall and subgroup analysis. The metaregression analysis did not identify the source of heterogeneity. In sensitivity analysis, heterogeneity reduced after one literature was excluded, but the pooled ORs did not alter remarkably, implying that the results were statistically stable and reliable. The Begg funnel plot and Egger's test were also negative for publication bias.

There were some limitations in our meta-analysis. First, our meta-analysis was based on only 13 studies including a small sample size; this may lead to a relatively low statistical power, especially in subgroup analysis. Second, not all the sources of controls were population based; this may induce deviations from the overall population. Third, no gene-environment or gene-gene interactions were considered which may be involved in the susceptibility to cancer. Fourth, the relation between R521K polymorphism and the chemosensitivity of anticancer drugs remains to be elucidated.

In conclusion, there is no significant association between EGFR R521K polymorphism and risk of cancer. However, further studies based on larger, stratified case-control populations and the effect of gene-gene, gene-environment, and the chemosensitivity of anticancer drugs are still needed to be evaluated.

## Figures and Tables

**Table 1:** Characteristics of studies included in the meta-analysis.

First author	Year	Country	Ethnicity	Cancer type	Genotyping Methods	Case						Control						HWE
						Number	GG	GA	AA	G	A	Number	GG	GA	AA	G	A	
Poole [11]	2011	Germany	Caucasian	Colorectal cancer	Mass Assay	2002	1122	743	137	2987	1017	2550	1456	925	169	3837	1263	0.18
Kallel [12]	2009	Tunisia	Arab	Breast cancer	PCR-RFLP	148	90	46	12	226	70	303	174	103	26	451	155	0.07
Wu [13]	2013	China	Asian	Hepatocellular carcinoma	TaqMan	402	84	205	113	373	431	643	157	316	170	630	656	0.69
Zhang [14]	2013	China	Asian	Gastric cancer	PCR-LDR	387	75	187	109	337	405	392	65	205	116	437	335	0.12
Choi [15]	2007	South Korea	Asian	Lung cancer	PCR-RFLP	582	206	279	97	691	473	582	215	268	99	698	466	0.32
Torres-Jasso [16]	2013	Mexico	Mexican	Gastric cancer	PCR-RFLP	72	31	33	8	95	49	100	49	43	8	141	59	0.94
Xie [17]	2012	China	Asian	Hepatocellular carcinoma	MALDI-TOF	311	69	130	112	268	354	311	55	144	112	254	368	0.46
Martinelli [18]	2011	Italy	Caucasian	Colorectal cancer	PCR-RFLP	70	36	23	11	95	45	72	24	38	10	86	58	0.47
Rebaï [19]	2009	Tunisia	Arab	Thyroid cancer	PCR-RFLP	106	59	41	6	159	53	302	174	98	30	446	158	0.06
Abdraboh [20]	2013	Egypt	Caucasian	Breast cancer	PCR-RFLP	64	11	50	3	72	56	86	48	36	2	132	40	0.14
Mustafa [21]	2013	Syria	Arab	Colorectal cancer	PCR-RFLP	47	19	27	1	65	29	48	20	21	7	61	35	0.76
Sobti [22]	2012	India	Indian	Breast cancer	PCR-RFLP	150	53	85	12	191	109	150	64	73	13	201	99	0.27
Hong [23]	2009	China	Asian	Breast cancer	TaqMan	2987	747	1460	780	2954	3020	2916	630	1516	770	2776	3056	0.19

**Table 2:** Meta-analysis of the R521K polymorphism with risk of cancer.

	*N*	AA versus GG				GA versus GG				AA versus GA + GG				AA + GA versus GG				A versus G			
		OR (95% CI)	*P*_*H*_	*I* ^2^ (%)	*P*	OR (95% CI)	*P*_*H*_	*I* ^2^ (%)	*P*	OR (95% CI)	*P*_*H*_	*I* ^2^ (%)	*P*	OR (95% CI)	*P*_*H*_	*I* ^2^ (%)	*P*	OR (95% CI)	*P*_*H*_	*I* ^2^ (%)	*P*
Overall	13	0.85 [0.74, 0.99]	0.25	19	0.12	1.04 [0.87, 1.25]	1∗10^−5^	74	0.67	0.99 [0.91, 1.08]	0.84	0	0.92	1.02 [0.86, 1.21]	0.0001	69	0.60	1.06 [0.95, 1.18]	0.0001	69	0.28
Cancer type																					
Breast cancer	4	0.94 [0.65, 1.38]	0.22	32	0.77	1.41 [0.77, 2.60]	1∗10^−5^	89	0.27	0.99 [0.88, 1.10]	0.88	0	0.80	1.40 [0.78, 2.50]	1∗10^−5^	89	0.26	1.18 [0.84, 1.64]	0.0007	83	0.33
Colorectal cancer	3	0.81 [0.42, 1.58]	0.18	42	0.98	0.85 [0.47, 1.55]	0.03	70	0.60	1.04 [0.83, 1.31]	0.97	0	0.72	0.85 [0.52, 1.38]	0.08	61	0.51	0.92 [0.72, 1.18]	0.21	35	0.78
Gastric cancer	2	0.94 [0.55, 1.59]	0.26	21	0.56	0.91 [0.61, 1.34]	0.26	21	0.48	0.97 [0.72, 1.30]	0.43	0	0.82	0.90 [0.52, 1.56]	0.11	60	0.71	1.43 [1.19, 1.72]	0.49	0	**0.001**
Hepatocellular carcinoma	2	1.01 [0.66, 1.57]	0.12	58	0.95	0.95 [0.57, 1.59]	0.05	73	0.85	1.05 [0.85, 1.30]	0.70	0	0.65	0.98 [0.61, 1.57]	0.06	73	0.92	1.03 [0.90, 1.18]	0.18	45	0.68
Other cancers	2	0.93 [0.61, 1.40]	0.27	17	0.75	1.12 [0.89, 1.40]	0.64	0	0.33	0.91 [0.68, 1.21]	0.23	30	0.52	1.07 [0.87, 1.32]	0.96	0	0.52	1.01 [0.87, 1.17]	0.67	0	0.90
Ethnicity																					
Arab	3	0.65 [0.34, 1.26]	0.29	19	0.13	1.05 [0.78, 1.42]	0.45	0	0.74	0.58 [0.34, 1.01]	0.91	0	0.06	0.97 [0.73, 1.28]	0.76	0	0.83	0.90 [0.72, 1.12]	0.87	0	0.35
Asian	5	0.91 [0.79, 1.05]	0.32	15	0.20	0.91 [0.76, 1.10]	0.06	57	0.35	0.99 [0.90, 1.09]	0.96	0	0.85	0.91 [0.76, 1.09]	0.04	60	0.30	1.06 [0.91, 1.25]	0.0004	80	0.44
Caucasian	3	1.18 [0.57, 2.45]	0.13	50	0.66	1.34 [0.43, 4.19]	1∗10^−5^	92	0.62	1.05 [0.84, 1.32]	0.75	0	0.65	1.40 [0.48, 4.09]	1∗10^−5^	92	0.54	1.21 [0.68, 2.15]	0.0005	87	0.51
Other ethnicities	2	1.15 [0.59, 2.27]	0.46	0	0.68	1.33 [0.91, 1.96]	0.72	0	0.14	1.09 [0.57, 2.07]	0.50	0	0.79	1.33 [0.92, 1.92]	0.86	0	0.13	1.18 [0.90, 1.55]	0.83	0	0.22

*N*: number of studies, OR (95% CI): odds ratio (95% confidence interval), and *P*_*H*_: *P* values of *Q*-test for heterogeneity test. Random effects model was used when *P*_*H*_ < 0.05 or *I* ^2^ ≥ 50%; otherwise fixed effect model was used.
